# Supplementary material for: Surface Microbiome Profiling of Dental Elevators Using Third‐Generation Sequencing: Implications for Infection Control in Dental Practice
Source: Microbiologyopen. 2025 Dec 8;14(6):e70178. doi: 10.1002/mbo3.70178 (PMC12685768; doi:10.1002/mbo3.70178)
Supplement: Supplementary file 1 — customer_backup. [file MBO3-14-e70178-s001.zip › customer_backup/customer_backup/taxa_summary/krona/groups/treat/M10.Krona.html]

Javascript must be enabled to view this page.

members
magnitude
magnitudeUnassigned

M10.krona

57454

57454

1

1

1

0

0

0

0

0

0

1

1

1

0

0

0

0

0

0

0

0

0

0

0

0

0

0

0

0

0

0

0

0

0

0

0

0

0

0

0

0

0

0

0

54

36

0

0

0

0

12

12

8

8

2

1

1

0

0

1

1

1

1

24

10

10

10

7

6

6

1

1

0

0

0

0

0

0

0

0

7

1

1

0

0

0

0

6

2

4

0

0

0

0

0

18

0

0

0

0

0

0

0

18

0

0

0

0

0

0

0

0

0

0

0

0

18

18

18

101

79

79

0

0

0

79

5

5

0

74

72

2

0

0

0

0

1

0

0

0

0

1

0

0

0

0

0

0

0

0

0

0

0

0

0

1

0

0

0

0

0

0

0

0

0

0

0

0

0

0

0

1

0

1

0

0

0

0

0

0

0

0

0

0

0

0

21

21

1

0

0

0

0

0

0

0

0

1

1

0

0

0

0

0

0

0

0

0

0

0

0

0

20

0

0

0

0

0

20

20

0

0

0

0

0

0

0

0

0

0

0

0

0

0

0

0

0

0

0

0

0

0

0

0

0

0

0

0

0

0

0

0

0

0

0

0

0

0

0

0

0

0

0

0

0

0

0

0

0

0

0

0

0

0

0

0

0

0

0

0

0

0

0

0

0

0

0

0

0

0

0

22

7

7

7

0

0

7

7

0

0

0

0

0

0

0

0

0

0

0

15

15

15

0

0

0

0

0

0

0

0

0

0

0

15

0

15

201

3

3

0

0

0

3

3

3

0

0

0

0

0

0

0

0

0

0

0

0

0

0

0

0

198

22

21

0

0

0

0

21

0

9

0

12

1

1

0

1

0

0

0

0

0

0

0

0

0

176

0

0

0

0

0

0

0

0

0

0

0

0

0

0

0

0

0

0

0

0

0

0

0

0

176

176

0

176

0

0

0

0

0

0

0

0

0

0

0

0

0

0

0

0

0

0

4348

197

0

0

0

0

3

0

0

0

3

0

0

0

0

0

0

0

0

3

0

3

0

0

0

0

2

2

1

0

1

0

0

1

1

0

0

0

0

0

0

0

0

0

0

0

0

0

0

0

0

0

0

0

0

0

0

0

0

0

0

0

0

0

0

0

0

0

0

46

46

0

0

6

0

0

0

6

0

0

0

0

0

0

0

1

1

0

0

0

0

0

0

0

0

0

0

0

0

0

0

0

0

12

0

8

1

3

0

0

0

25

0

0

0

0

0

0

0

8

0

0

0

0

3

0

0

7

0

1

1

0

0

0

0

1

0

0

0

0

4

0

2

0

0

0

2

0

0

0

0

0

0

0

0

0

131

0

0

0

0

0

0

0

0

0

0

0

0

0

0

0

0

0

0

0

0

0

0

0

0

0

0

0

0

0

0

0

0

0

0

0

0

0

0

0

0

0

0

0

0

0

0

0

0

0

0

0

74

0

0

0

0

0

0

0

39

39

0

0

0

0

1

1

0

0

0

0

0

1

1

2

0

1

0

0

1

0

0

0

0

31

0

0

0

0

0

0

0

0

0

7

21

3

0

0

0

0

0

0

0

0

0

0

0

0

0

0

0

0

0

0

0

0

0

0

0

0

0

0

0

0

0

0

0

0

0

0

0

0

0

0

0

0

1

1

1

0

0

0

0

7

0

0

0

0

0

2

2

5

5

0

0

0

0

0

0

0

0

0

0

0

0

0

0

0

0

49

1

1

0

0

0

0

0

0

0

0

0

0

7

0

0

0

0

4

0

0

0

0

0

3

0

0

0

0

0

15

13

2

0

0

0

0

0

0

0

0

26

26

0

0

0

0

0

0

0

0

0

0

0

0

0

0

0

0

0

0

0

0

0

0

0

0

0

0

0

0

0

0

0

0

0

0

0

0

1

1

0

0

0

0

1

1

0

0

0

0

0

0

0

0

0

0

0

0

0

0

0

0

0

0

0

0

0

0

0

0

0

0

0

0

0

0

0

0

0

0

0

0

0

0

0

4

4

0

0

0

0

0

2

2

1

1

0

0

0

0

0

0

0

0

0

0

0

0

0

0

0

1

0

0

1

0

0

0

0

0

0

0

0

0

0

0

0

0

0

0

0

0

0

0

0

0

0

0

0

0

0

0

0

0

0

0

0

0

0

0

0

10

10

0

0

0

0

0

0

0

0

0

0

0

0

0

0

0

0

0

0

0

0

0

0

0

1

1

0

0

0

0

0

0

0

2

1

0

0

0

1

4

0

0

4

0

0

0

0

3

0

3

0

4151

0

0

0

0

0

0

0

0

0

0

0

0

0

0

0

0

0

0

0

2318

1

0

0

1

1

0

0

0

0

0

0

0

0

0

0

0

0

0

0

0

0

0

0

0

0

0

0

0

0

0

0

0

0

0

0

558

0

0

0

0

500

500

53

1

14

27

11

5

0

1

0

0

0

3

0

0

0

1

0

0

0

0

0

0

0

0

0

0

0

0

0

0

0

0

0

0

0

0

0

0

0

0

0

60

0

0

0

0

0

0

3

0

0

3

52

1

0

51

5

5

1019

97

97

0

0

92

65

27

830

22

123

685

0

0

0

499

0

0

0

0

0

0

0

0

0

0

0

0

0

0

0

0

1

0

1

0

0

18

0

18

0

0

0

0

0

133

0

133

0

2

2

0

0

0

345

345

0

0

181

0

0

181

1

180

3

0

0

0

0

0

0

0

0

3

3

1

2

0

0

0

0

0

0

0

0

0

0

0

0

0

0

0

26

26

26

0

26

0

0

0

0

1

1

1

0

1

0

0

0

0

0

0

0

0

0

0

73

0

0

0

72

1

1

0

0

0

0

0

0

0

71

0

0

0

71

0

0

0

0

0

0

0

0

0

0

0

0

0

0

0

0

0

0

0

0

0

1

1

0

0

1

0

0

0

0

0

0

0

0

0

0

0

0

0

0

0

0

0

0

0

0

0

0

0

0

0

0

0

0

0

0

0

0

0

0

0

0

0

0

0

0

0

0

0

1

0

0

0

1

0

0

0

0

1

0

1

0

0

0

0

1717

0

0

0

0

0

1

1

1

0

0

0

0

0

0

0

0

0

8

3

3

0

0

5

5

0

0

28

28

28

0

7

7

7

0

0

0

2

2

2

1671

0

0

0

0

0

0

85

85

245

0

8

7

230

1341

1261

80

0

0

0

0

0

0

0

0

0

0

0

2

2

2

2

0

0

0

0

0

0

0

0

0

0

0

0

0

0

0

0

0

0

9

0

0

0

0

0

0

0

0

0

0

0

0

0

9

0

0

0

0

0

0

0

0

0

2

1

0

1

0

7

0

0

0

0

0

0

0

0

0

0

0

7

0

0

0

0

0

0

0

0

0

0

0

0

0

0

0

0

0

0

0

0

0

0

0

1

1

0

0

0

0

0

0

0

0

0

0

0

0

1

0

0

1

0

0

0

0

0

0

0

0

0

0

0

0

0

0

0

0

0

0

0

0

0

0

0

0

0

0

0

0

0

0

0

0

1

0

0

0

0

0

0

0

0

0

0

0

0

0

0

0

0

0

0

0

0

0

0

0

0

0

1

0

0

0

0

0

0

0

0

0

0

0

0

0

0

0

0

0

1

1

0

0

0

0

0

0

0

0

0

1

1

0

0

0

0

0

0

0

0

0

0

0

0

0

0

0

0

0

0

0

0

0

0

0

0

0

0

0

0

0

0

0

0

0

0

0

0

0

0

0

0

0

0

0

0

0

0

0

0

0

0

0

0

0

0

0

0

0

0

0

0

0

0

0

0

0

0

0

0

0

0

14360

0

0

0

0

0

14360

19

0

0

0

19

0

0

0

1

0

1

0

0

0

0

0

0

0

0

0

0

0

0

0

0

7

0

0

4

0

0

3

0

0

0

0

0

0

0

0

0

0

0

0

0

0

0

0

0

0

0

0

0

0

0

0

0

11

11

0

0

0

0

0

0

0

0

31

0

0

0

25

0

0

0

0

0

0

1

1

0

0

0

0

0

0

0

0

0

0

0

0

0

24

7

0

0

0

6

11

0

0

0

0

0

0

0

0

0

0

0

0

0

0

0

0

0

0

0

0

0

0

0

0

0

0

6

6

6

0

0

0

0

0

0

14284

0

0

0

0

0

0

0

0

0

0

0

10346

20

20

30

30

515

515

9781

9781

808

0

0

0

0

0

0

0

0

0

0

0

1

1

807

1

0

0

0

0

15

0

729

62

0

0

0

0

0

0

0

0

0

0

0

0

0

1

0

0

1

1

0

0

0

2368

2368

0

0

0

1

2

96

23

0

0

0

2097

1

0

147

0

0

1

20

0

0

19

0

19

1

1

0

0

0

0

0

640

0

0

0

15

0

15

121

0

121

0

0

0

315

315

1

1

0

0

108

0

0

7

0

49

0

0

0

0

0

0

0

0

0

0

0

52

80

0

0

0

0

0

0

0

0

10

0

0

0

6

64

0

0

0

0

0

0

0

0

71

71

0

11

25

35

0

0

0

0

0

30

30

30

1

0

0

0

1

0

0

0

0

1

1

0

0

0

0

0

0

0

0

0

0

0

0

0

0

0

25

3

0

0

0

0

0

0

3

0

1

2

0

0

0

0

0

0

0

0

0

0

0

10

0

0

0

0

0

0

0

0

0

10

0

10

0

0

0

0

0

0

0

0

0

0

0

0

12

0

0

0

0

0

0

0

0

0

0

0

0

0

0

0

0

0

12

0

0

0

0

1

2

3

0

0

5

1

0

0

0

0

0

0

0

0

0

0

0

0

0

0

0

0

0

0

0

0

0

0

0

0

0

0

0

0

0

0

0

0

0

0

0

0

0

1

1

0

0

0

0

0

0

0

0

0

0

0

0

0

0

0

0

0

0

0

0

0

0

0

0

0

0

0

1

1

1

1

0

0

0

0

0

0

0

0

0

0

0

0

0

0

0

0

0

0

0

0

0

0

0

0

0

0

0

0

0

0

888

427

427

0

0

0

126

22

22

0

0

0

104

0

0

104

0

301

4

4

208

10

7

25

166

0

0

0

0

7

0

7

0

0

37

31

6

0

0

0

0

45

0

0

45

0

0

0

0

0

0

0

26

18

0

0

0

0

0

0

0

0

0

0

0

0

0

0

0

18

18

0

18

0

0

0

0

0

0

0

0

0

0

0

0

0

0

0

0

0

0

0

1

0

0

0

0

0

0

0

0

0

0

0

0

1

1

1

0

0

0

0

0

0

0

7

3

2

2

0

1

0

1

0

0

0

0

1

1

0

1

0

2

0

0

0

0

0

0

2

1

1

1

0

0

0

0

1

1

9

2

0

0

0

0

0

0

0

0

0

0

0

0

0

0

0

0

0

0

0

0

0

0

0

0

0

0

0

2

0

0

1

1

1

1

0

0

0

0

7

7

7

7

0

0

0

0

0

0

0

0

0

424

0

0

0

0

0

0

0

0

0

0

0

0

0

0

0

0

0

0

0

0

1

1

0

0

0

0

0

0

0

0

0

0

0

0

0

0

0

0

0

0

1

0

1

0

0

0

0

0

0

0

0

0

0

0

0

0

0

0

0

0

0

0

20

7

7

3

1

0

3

0

0

0

0

0

0

0

0

13

9

3

6

0

0

4

4

0

0

11

11

0

0

0

11

3

5

0

0

0

2

0

1

0

0

5

0

0

0

0

0

0

0

0

0

0

0

0

0

0

0

0

0

0

0

0

0

0

0

5

0

0

3

3

2

2

0

0

0

0

0

0

0

0

0

0

0

0

0

58

58

0

0

0

0

58

1

0

2

0

0

8

0

0

0

7

20

13

7

0

0

0

0

0

0

0

0

0

0

0

0

0

0

0

0

0

0

0

177

171

0

0

0

0

15

15

0

0

0

0

0

0

0

0

0

0

156

0

156

0

0

0

6

1

0

0

1

0

0

5

0

1

0

0

0

0

0

0

0

0

0

0

0

0

1

0

0

0

0

3

0

0

0

0

0

0

0

0

0

0

3

3

0

0

0

0

3

2

0

1

0

0

0

0

0

0

0

0

0

0

0

0

0

0

0

0

0

0

0

0

0

149

72

0

0

47

47

14

2

12

0

0

0

0

0

9

0

3

1

0

5

1

1

0

0

1

1

3

0

0

1

1

0

0

2

0

2

0

0

0

0

0

0

0

0

0

0

3

3

3

0

0

0

0

60

2

2

0

0

0

0

5

4

1

0

0

0

0

0

0

0

0

0

0

0

0

0

0

25

25

28

0

7

21

0

0

0

0

0

0

0

0

0

0

0

11

0

0

11

11

2

2

2

2

0

2

0

0

0

0

0

0

0

0

0

0

0

0

0

0

0

0

0

0

0

0

0

0

0

0

0

0

0

0

0

0

0

0

0

0

0

0

0

0

0

0

0

0

0

0

0

0

1

1

1

1

1

1

781

781

781

752

3

3

0

0

0

0

749

595

154

29

29

0

0

6

0

0

23

0

0

0

29

29

29

0

0

0

0

0

0

0

28

28

0

28

0

0

0

0

0

0

0

1

1

1

1

0

0

0

0

0

0

0

0

0

0

0

0

0

0

0

1

1

0

0

0

0

0

0

1

1

1

1

1

1

1

1

1

0

0

0

16

0

0

0

0

0

0

0

0

0

0

0

0

0

0

0

0

0

0

0

0

0

0

0

0

0

0

0

0

0

0

0

0

0

0

0

0

0

0

0

0

0

0

0

0

0

0

0

0

0

0

0

0

0

0

0

0

0

0

0

13

0

0

0

0

13

13

13

13

0

0

0

0

3

3

3

0

0

3

0

0

0

0

0

0

0

3

0

0

0

0

0

0

0

0

0

0

0

0

0

0

0

0

0

0

0

0

280

0

0

0

0

0

0

0

0

0

0

0

0

0

0

0

0

0

0

0

0

0

0

0

0

0

0

0

0

0

0

0

0

0

0

0

0

0

0

0

0

0

0

0

0

82

82

82

82

82

0

0

0

0

0

0

0

0

0

0

0

0

0

0

0

0

0

0

0

198

198

32

32

32

0

0

0

93

93

93

0

0

0

0

0

0

0

0

73

25

25

0

0

0

17

17

0

0

22

22

9

9

0

0

0

0

0

0

0

0

0

0

0

0

0

0

0

0

0

0

0

0

0

207

207

207

207

0

0

207

101

106

0

0

0

0

0

0

0

0

0

0

0

0

0

0

0

0

0

0

0

0

0

0

0

0

0

0

0

0

0

0

0

0

0

0

0

0

0

0

0

0

0

0

0

0

0

0

0

0

0

0

0

0

0

0

0

0

0

0

0

0

0

0

0

0

0

0

0

0

0

0

0

0

0

0

0

0

0

0

0

0

0

0

0

35498

26082

40

40

0

0

0

40

40

0

0

0

0

0

0

0

155

155

7

7

148

148

1

0

0

0

0

0

0

1

1

1

0

0

34

34

34

1

1

32

19508

19508

0

0

0

0

0

0

0

0

0

20

3

17

0

0

0

147

5

142

0

0

0

0

0

0

0

0

55

55

82

82

0

0

0

0

14411

14264

143

4

0

0

0

0

0

0

235

3

1

1

5

0

0

0

0

10

158

0

0

0

41

0

16

0

0

0

0

0

0

0

0

2

0

2

0

0

10

0

8

2

46

1

2

0

42

1

0

2269

29

0

0

0

1759

44

423

0

14

0

0

1830

0

0

160

1669

1

16

16

0

0

0

0

14

14

0

0

0

222

9

213

0

0

2

2

0

0

9

0

0

9

0

0

0

0

0

6

6

0

0

0

0

7

0

0

7

72

72

1

0

1

0

0

0

52

0

32

0

6

0

11

1

0

0

0

1

1

0

0

0

0

0

0

0

0

0

0

1739

919

81

81

210

0

17

193

13

0

13

0

0

180

180

0

312

312

95

0

13

0

81

1

28

28

0

0

0

554

12

12

0

0

192

192

0

0

301

301

0

0

49

49

0

266

0

0

0

0

0

0

0

0

0

266

266

0

0

73

73

0

0

0

0

50

45

0

0

5

0

0

0

23

23

507

28

28

28

0

0

0

0

0

0

76

76

76

403

403

403

0

0

0

0

0

0

0

0

0

3588

2471

62

0

62

0

0

1

1

31

0

31

528

528

9

9

97

27

0

47

23

0

0

5

5

18

0

0

18

0

67

0

0

63

4

0

1120

448

22

24

0

382

85

0

0

159

0

0

0

0

0

8

0

8

525

135

0

0

390

64

64

64

0

0

0

7

2

2

0

5

1

4

0

0

0

0

0

0

0

32

32

32

0

0

0

0

0

1006

130

0

0

71

0

0

59

6

6

12

12

320

196

89

35

50

7

43

1

0

0

0

1

454

370

84

33

33

0

0

0

8

8

8

437

145

145

145

9

9

9

252

252

252

0

0

0

31

31

31

8258

0

0

0

0

62

36

0

0

0

0

0

0

2

0

2

0

0

0

1

0

1

33

33

0

0

0

0

0

0

26

0

0

0

0

0

0

0

26

1

0

0

0

1

1

0

1

0

13

1

6

1

0

0

1

0

0

0

0

0

0

0

0

0

0

0

0

0

0

1337

397

0

0

33

33

1

1

362

1

9

0

350

2

0

0

0

0

0

1

0

1

0

0

940

140

140

65

65

0

0

0

0

0

0

671

671

0

0

0

9

9

40

40

0

0

0

0

0

15

15

0

0

0

0

0

0

0

0

0

0

0

0

0

0

1017

159

0

0

0

0

159

45

114

858

858

858

0

0

1

1

1

1

0

13

13

0

0

0

0

13

1

0

0

0

1

0

0

0

0

0

0

0

0

0

0

9

0

0

0

1

0

1

0

0

0

0

0

0

0

0

0

0

0

0

600

114

114

114

6

6

6

243

243

243

237

237

237

6

6

6

5

1

5150

2

2

2

0

321

0

0

0

0

319

319

2

2

0

0

4406

4404

0

238

25

0

3496

0

101

2

539

0

1

2

0

2

2

0

3

3

0

3

418

0

0

0

0

9

4

3

2

0

0

0

0

84

0

32

1

0

0

51

0

0

35

35

0

0

0

0

0

8

8

279

0

0

279

1

1

0

1

1

1

1

0

0

0

0

0

0

0

0

0

0

0

0

0

0

0

0

0

0

0

0

72

72

0

0

72

72

0

0

0

0

0

0

0

0

0

0

0

0

0

0

0

0

0

1158

1

1

0

0

1

1

1157

0

0

0

206

0

0

0

0

0

0

0

0

0

206

117

0

0

0

0

0

0

10

79

951

711

702

9

69

69

171

0

139

32

0

0

0

0

0

0

0

0

0

0

0

0

2

0

0

0

0

0

0

0

0

0

0

0

0

0

0

0

0

0

0

0

0

0

0

0

0

0

0

0

0

0

0

0

0

0

0

0

0

0

0

0

0

2

0

0

0

0

2

2

0

0

0

0

0

0

2

2

0

0

0

0

0

0

0

0

0

0

0

0

0

0

0

0

0

0

0

0

0

0

0

0

0

0

0

0

0

0

661

661

661

661

661

0

661

0

0

0

0

0

0

0

0

0

0

0

0

0

0

0

0

0

0

0

0

0

0

0

0

0

0

0

0

0

0

0

0

0

0

0

0

0

0

0

0

0

0

0

0

0

0

0

0

0

0

0

0

0

0

0

0

0

0

0

0

0

0

0

0

0

0

0

0

0

0

0
